# Chemical proteomics tracks virus entry and uncovers NCAM1 as Zika virus receptor

**DOI:** 10.1038/s41467-020-17638-y

**Published:** 2020-08-04

**Authors:** Mayank Srivastava, Ying Zhang, Jian Chen, Devika Sirohi, Andrew Miller, Yang Zhang, Zhilu Chen, Haojie Lu, Jianqing Xu, Richard J. Kuhn, W. Andy Tao

**Affiliations:** 1 Department of Chemistry, Purdue University, West Lafayette, IN 47907 USA; 2 Institutes of Biomedical Sciences and NHC Key Laboratory of Glycoconjugates Research, Fudan University, Shanghai, 200032 China; 3 Department of Biochemistry, Purdue University, West Lafayette, IN 47907 USA; 4 Shanghai Public Health Clinical Center, Fudan University, Shanghai, 200032 China; 5 Department of Biological Sciences, Purdue University, West Lafayette, IN 47907 USA; 6 Purdue Institute of Inflammation, Immunology and Infectious Disease, Purdue University, West Lafayette, IN 47907 USA

**Keywords:** Proteomics, Chemical tools, Virus-host interactions

## Abstract

The outbreak of Zika virus (ZIKV) in 2016 created worldwide health emergency which demand urgent research efforts on understanding the virus biology and developing therapeutic strategies. Here, we present a time-resolved chemical proteomic strategy to track the early-stage entry of ZIKV into host cells. ZIKV was labeled on its surface with a chemical probe, which carries a photocrosslinker to covalently link virus-interacting proteins in living cells on UV exposure at different time points, and a biotin tag for subsequent enrichment and mass spectrometric identification of the receptor or other host proteins critical for virus internalization. We identified Neural Cell Adhesion Molecule (NCAM1) as a potential ZIKV receptor and further validated it through overexpression, knockout, and inhibition of NCAM1 in Vero cells and human glioblastoma cells U-251 MG. Collectively, the strategy can serve as a universal tool to map virus entry pathways and uncover key interacting proteins.

## Abstract (web summary)

The mechanism underlying the cellular entry of Zika virus is not fully understood. Here, the authors use a chemically modified virus and time-resolved proteomics to capture interacting host proteins during virus entry and identify NCAM1 as a ZIKV receptor.

## Introduction

Zika virus (ZIKV) has been the focus of immense investigation since the recent epidemic. While studies on ZIKV structure revealed its structural similarity to other virus relatives within the family *Flaviviridae*, some notable differences such as the Asn154 glycosylation site present on each of the E proteins may affect receptor interactions, antibody response, and downstream biology of the virus^1,2^. For the past several years, efforts have been made to understand fundamental biology of ZIKV infection, including the use of animal models to evaluate their immune response to the virus invasion^3^, as well as potential therapeutics against ZIKV infection^4–6^. However, unanswered questions remain regarding molecular mechanisms of host restriction and immune evasion^7^. Identification of direct interactors of ZIKV during its entry into host cells could not only suggest the molecular pathways manipulated by the virus, but also provide immense opportunity to develop antivirals by offering new potential drug targets. Owing to the transient nature of these interactions and the extreme rapidness in the flavivirus entry in general, identification of dynamic interactors of virus is a formidable task.

Our understanding of ZIKV internalization and cellular trafficking would greatly benefit from a systematic, temporal characterization of major proteins involved in the dynamic virus entry. Real-time fluorescence microscopy has been used to study the transport, acidification, and fusion of single virus^8,9^. The movement of single virus demonstrated an intriguing and dynamic process. The molecular information, in particular protein machinery involved in the process, is typically limited to labeled molecules in the amazing technique. On the other hand, affinity and chemical proteomics studies identified virus-interacting proteins^10–12^. The molecular mechanisms and dynamic virus–host interactions responsible for the internalization of ZIKV, however, have remained unresolved. A systematic quantitative measurement of temporal changes in virus–protein interactions may prove extremely valuable for the identification of host molecules as potential therapeutic targets. We have previously used chemical proteomics strategies and modified a nanoparticle, polyamidoamine generation 3 dendrimer, to understand the endocytic pathways of a nanoparticle^13^ followed by a recent study to investigate the entry of *Salmonella* into host cells^14^. Here, we expand the concept and hypothesize that chemical modification of ZIKV would not significantly affect its infectivity and would allow us to track the virus entry into living cells and identify virus-interacting proteins by mass spectrometry (MS), revealing the spatiotemporal distribution of the key proteins involved in the pathways for ZIKV entry and trafficking.

## Results

### Synthesis and characterization of ZIKV-labeling probe

**Fig. 1:**
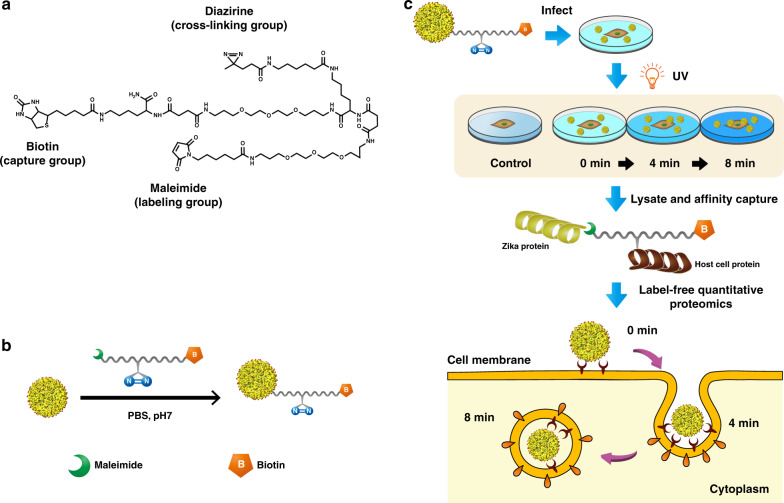
Chemically labeling ZIKV surface E proteins and capturing virus-interacting proteins. **a** Structure of virus-labeling reagent. Maleimide reacts with available cysteines on the virus surface under mild conditions, diazirine enables crosslinking host proteins at fixed time points allowing tracking virus movement in real time, and biotin acts as a handle for protein enrichment and identification by downstream mass spectrometric analysis. The three functionalities are separated by a membrane-impermeable polyethylene glycol (PEG)-like linkers, while offering the flexibility required for capturing interacting proteins with the aqueous solubility. **b** Labeling of ZIKV surface proteins. Purified Zika virions were diluted and reacted with the labeling reagent in PBS at 4 °C. Reaction was quenched with threefold excess cysteine for 1 h. **c** Workflow for capturing virus receptors and tracking its cellular entry. Labeled ZIKV was diluted in DMEM and incubated with confluent cells for 1 h at 4 °C. In addition, cells were incubated with the labeled viruses in 37 °C for fixed time points to allow virus entry. Unbound viruses were removed and cells were directly exposed to UV light. Cells were lysed and biotinylated proteins were captured on the avidin beads. Proteins were digested on beads using sequential Lys-C and trypsin digestion, and analyzed by LC–MS/MS. Label-free quantitation was performed using MaxQuant to identify and quantify the crosslinked proteins.

We devised and synthesized a multifunctional chemical probe (Fig. 1a; Supplementary Figs. 1–3) bearing a labeling group that conjugates the probe to the ZIKV surface, a photo-reactive group that allows for covalent crosslinking of ZIKV proteins to interacting host cell proteins upon UV exposure, and an isolation tag of biotin for purifying the interacting proteins for quantitative MS analysis, thus facilitating the investigation of host–pathogen interactomes in a time-resolved manner (Fig. 1b). We chose the maleimide group to label the virus through its specific conjugation with thiol groups on the virus surface proteins at physiological condition to form a stable thioether linkage. As sulfhydryls thiols are present in most proteins but are not as abundant as primary amines, we expected limited labeling on cysteine residues would have a minimal labeling effect on the ZIKV activity. According to the structure of mature ZIKV determined by cryo-electron microscopy by our group^2^, there are 13 cysteines in ZIKV E (12 in the ectodomain, 1 in the transmembrane domain) and no cysteine in M protein (Supplementary Fig. 4a; cysteine residues are highlighted as gray spheres in the structures). In addition, ZIKV, like other flaviviruses and enveloped viruses in general, is quite unstable and prone to undergo structural changes under external influence^15^. Considering the virus stability and infectivity, we preferred minimal labeling of virus through the maleimide-thiol conjugation under mild conditions at neutral pH. Moreover, the three functionalities are separated by a polyethylene glycol (PEG)-like linker to improve water solubility, while offering the flexibility for efficient crosslinking and enrichment.

The labeling was first examined with a standard protein and then with intact ZIKV. Bovine serum albumin (BSA) was incubated with 1 mM of reagent in phosphate buffer pH 7 at 4 °C and the labeled protein was enriched on streptavidin beads and analyzed by SDS-PAGE. The labeling efficiency was estimated to be 25–30% (Supplementary Fig. 4b). After labeling, modified ZIKV was lysed, and the labeled ZIKV surface proteins were purified on streptavidin beads and assessed by silver stain and western blotting using the 4G2 antibody against the E protein (Supplementary Fig. 4c). We further analyzed the captured proteins using MS. Multiple unique peptides from E protein were identified by MS (Supplementary Fig. 5). We did not identify any peptide from virus membrane (M) protein, capsid (C), or any of the nonstructural proteins of ZIKV, further confirming the exclusive tagging of virus surface with the reagent. This result is primarily owing to the membrane impermeable attribute of the chemical proteomic probe imparted by the PEGylated linkers. Finally, we examined the effect of labeling purified ZIKV with several concentrations and labeling time points by the plague assay. No loss of infectivity was observed under labeling conditions for 1 mM reagent concentration (Supplementary Fig. 4d). Hence, we concluded that the minimal labeling of virus achieved by cysteine-reactive maleimide group does not perturb the infectivity of the virus.

### Chemical proteomics to track the early-stage entry of ZIKV

We used the labeled ZIKV to infect Vero cells and interacting proteins were crosslinked at fixed time points to identify the virus–host factors and elucidate the virus entry mechanism (Fig. 1c). Flavivirus are quite promiscuous in their selection of receptors for entry to different cells. The complex entry mechanism might involve multiple receptor interactions to help virus internalize. Though some previous studies have identified AXL, a TAM family tyrosine kinase, as a putative receptor for ZIKV^16^, some conflicting evidence including ours suggests that the virus might employ multiple different classes of receptors for entry^4,17^. Furthermore, while most of the viruses are believed to enter cells by clathrin-mediated endocytosis, there is no evidence suggesting the absence of any parallel mode of virus entry. The complexity of the flavivirus entry mechanism hints at the presence of varied virus–protein interactions after membrane recruitment of host cellular proteins to initiate viral infection. In order to identify ZIKV receptors, we allowed the virus to attach to the cells at 4 °C for 1 h, followed by UV photo crosslinking on ice. For the virus entry, we chose 4 and 8 min according to a previous study that indicates the membrane fusion of a similar flavivirus at 512 s post binding, during its entry into Vero cells^9^. After crosslinking proteins at designated time points of attachment or entry, cells were harvested and proteins were extracted, followed by the enrichment using avidin beads. We reasoned that the chemical proteomic probe on the virus surface only crosslinks with proteins in direct contact with ZIKV, which can subsequently withstand vigorous washing conditions to remove nonspecifically bound proteins. The tryptic peptides derived from enriched samples were then analyzed by nanoflow HPLC coupled to high-resolution MS. Proteins were identified by a shotgun proteomic strategy and quantitated using the label-free method to measure their relative abundance across three time points and to distinguish crosslinked proteins from nonspecifically bound proteins.

**Fig. 2:**
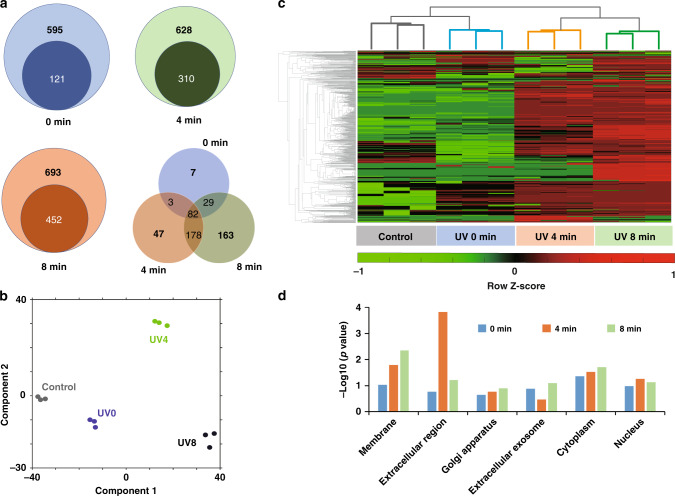
Proteomic analysis of crosslinked proteins. **a** Proteins crosslinked at different time points of ZIKV infection. Venn diagram showing the number of proteins crosslinked at each time point; proteins with a ratio of 2 and above compared with control were only considered for analysis. Detailed list of these proteins were shown in Supplementary Data 1. **b** Principal component analysis (PCA) of proteins among different time points. **c** Heatmap demonstrating log protein intensities at 0, 4, and 8 min of infection normalized to the control. **d** Gene Ontology cellular component analysis of crosslinked proteins using DAVID, highlighting membrane along with *p* values. Detailed list of the proteins is shown in Supplementary Data 1.

In total, we identified around 500 crosslinked proteins across three time points, out of which more than 70 proteins are previously implicated in virus infection (Fig. 2a and Supplementary Data 1). The principal component analysis (PCA) shows that all biological replicates are tightly clustered together and each time point is well-separated, meaning the samples are clustered by the nature of sample but not the batch (Fig. 2b). The PCA and heatmap analysis also indicates that distinctive proteins were crosslinked at three different time points, suggesting that the strategy was able to reveal the temporal distribution of the interacting proteins crosslinked with ZIKV during the virus’ early entry (Fig. 2b, c). Gene ontology (GO) analysis, as expected, indicated proteins annotated as membrane and extracellular region were significantly overrepresented in the crosslinked proteins across all time points (Fig. 2d). To further investigate whether the strategy was also capable of correlating spatial information with the virus crosslinked proteins, we performed the STRING analysis to determine whether there is statistical overrepresentation of specific genes or proteins in the sample at specific time points and identify proteins specific at the attachment or cellular entry stages (Supplementary Fig. 6).

Notably, we identified 121 crosslinked proteins at 0 min, 24 of which were membrane proteins, such as C1QBP, CD9, ITGA3, LETM1, NCAM1, RACK1, and SLC3A2. Complement C1q binding protein is a key host factor for efficient respiratory syncytial virus (RSV) production^18^. The tetraspanin CD9 facilitates MERS-coronavirus entry by scaffolding host cell receptors and proteases^19^. E-Syt proteins were discovered to impact the formation of virus-induced syncytia during HSV-1 infection^20^. Integrin α3 directly interacts with hepatitis E virus (HEV) and plays a key role in cellular attachment and entry of neHEV^21^. Receptor of activated protein C kinase 1 (RACK1) is important for lymphocystis disease virus entry and infection^22^. 4F2 cell-surface antigen heavy chain (SLC3A2) was reported interacting with ZIKV NS4B protein and was indicated as a candidate host factor for ZIKV infection^12^. In this study, co-immunoprecipitation followed by western blotting of transduced cells verified that SLC3A2 specifically associates with ZIKV ENV (Supplementary Fig. 7a). Interestingly, neural cell adhesion molecule (NCAM1), which was reported as a receptor for rabies virus^23^, was identified at different time points and presents as a candidate receptor for ZIKV infection. Previous analysis of the global proteomic changes that occurred during differentiation of hNPCs into neurons also revealed significant upregulation of NCAM1^12^. Moreover, a few other noticeable proteins crosslinked specifically at 0 min include AP2M1 and CALML5. AP2M1 is the µ2 subunit of adaptor protein-2 (AP-2) complex that recognizes the tyrosine-rich sorting signals on the cytoplasmic tail of receptor proteins^24^. Our crosslinking experiment validates the complex’s structural assembly on the membrane as AP2M1 was selectively crosslinked at 0 min of infection, while no other AP-2 subunits were observed. AP2M1 has further been shown to modulate early-stage infectious entry of HCV, in its phosphorylated form^25^. CALML5 was discovered as a potential host factor for ZIKV infection^12^.

**Fig. 3:**
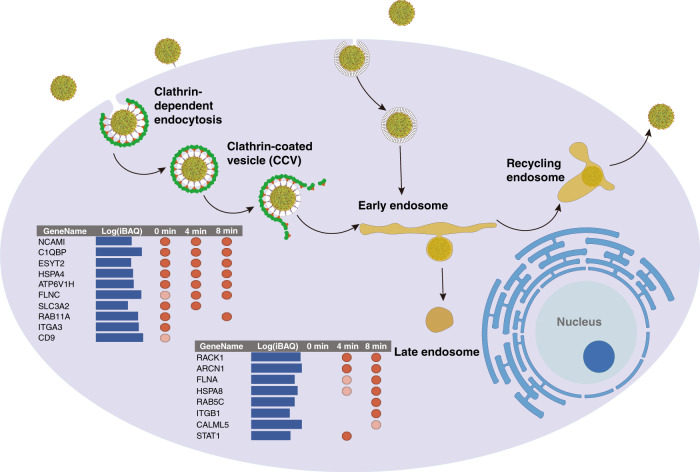
Proposed endocytosis pathways of ZIKV entry into host cells. Temporal illustration of ZIKV-crosslinked proteins involved in major endocytic mechanisms for ZIKV internalization. Listed are proteins crosslinked at different time points of infection. Blue bar indicated log(iBAQ) intensity. Red dot denoted crosslinked proteins at specific time point, dark red indicated log2-fold change between samples and control >2, and light red indicated log2-fold change between samples and control is between 1 and 2. A detailed list of these proteins with *p* value is shown in Supplementary Data 2.

We further demonstrated that the strategy allowed us to identify clusters of proteins representing a temporal shift of ZIKV subcellular localizations and the functions of crosslinked proteins are highly correlate to the temporal information in many cases (Fig. 3, Supplementary Data 2). For example, at 4 min, we observed certain proteins enriched such as STAT1, a key mediator of type-I interferon signaling. It has been reported that ZIKV suppresses the host immune response by inhibiting the type-I interferon signaling pathway^26^. Previous studies also have suggested the mechanism of blocking immune response by ZIKV as either the degradation of signal transducer and activator of transcription (STAT2)^27^, or antagonism of STAT1 and STAT2 phosphorylation^28^. RACK1, which mediates the interactions between IFN receptor and STAT1, was also identified as a ZIKV-interacting protein at that time point. Previously, a different class of virus was shown to interact with RACK1, thus initiating the dissociation of RACK1–STAT1 complex and inhibition of interferon signaling by the virus^29^. Our study emphasizes the involvement of both STAT1 and RACK1 in the immune response elicited by the ZIKV infection, suggesting ZIKV might employ a similar approach to suppress the host immune response.

We also identified an isoform of a key component of recycling endosomes, RAB11A. The role of RAB11A in transport of viral ribonucleoprotein or core proteins to plasma membrane for the generation of new virus particles of influenza and Hepatitis C Virus (HCV), respectively, is well documented^30^. However, some viruses may also utilize the recycling endosomal pathway to evade lysosomal degradation. Our study suggests the use of the recycling pathway by ZIKV after entry. Besides RAB11A, we also observed RAB5C at 8 min. Previous studies have established the importance of RAB5C in the entry of ZIKV^16^. The identification of RAB5C and RAB11A in our chemical proteomics experiment at a late time point of entry is consistent with the published data on Japanese Encephalitis Virus (JEV)^31^, a neurovirulent pathogen from the flavivirus genus structurally similar to the ZIKV. Other proteins identified of infection include HSPA8, also known as heat shock cognate 70 (Hsc70), known for its role in vesicle uncoating in the later stage of endocytosis^32^. Overall, more than 50 direct interactors such as AP2B1 and ITGB1 were also identified as ZIKV interactors in previous work^33^, further indicating the confidence of the identified interactors in our study.

In addition, we examined temporally ZIKV-crosslinked proteins against protein components involved in major endocytic mechanisms for ZIKV internalization. Prior studies showed ZIKV infection could be prevented by lysosomotropic agents which neutralize the normally acidic pH of endosomal compartments and was also blocked by chlorpromazine^34,35^, indicating the requirement of clathrin-mediated endocytosis and low pH for ZIKV infection. In our study, ITGB1, HSPA8, RAB5C, RACK1, and RAB11A were crosslinked, suggesting the clathrin-mediated pathway employed by the virus to infect Vero cells. Furthermore, identification of ARCN1, ITGA3, FLNA, and FLNC also suggests the utilization of a caveolar-mediated pathway by ZIKV for endocytosis.

### Identification and verification of NCAM1 as ZIKV receptor

**Fig. 4:**
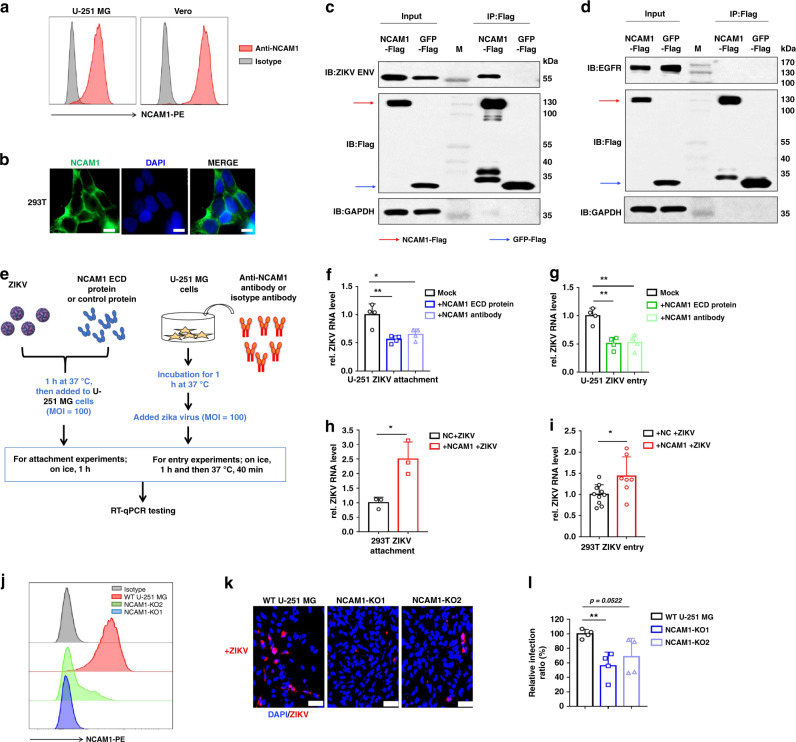
Identification and validation of NCAM1 as a potential host receptor for ZIKV. **a**, **b** Flow cytometry (**a**) and immunofluorescence (**b**) analyses of NCAM1 expression in U-251 MG, Vero or 293T cells. 293T cells were transfected with NCAM1 for 48 h. The length of scale bars in (**b**) is 10 μm. **c**, **d** Immunoblot (IB) and immunoprecipitation (IP) of lysates of ZIKV ENV (**c**) or EGFR (**d**) and Flag-NCAM1 or Flag-GFP overexpressed 293T cells. **e** Schematic representation of the viral binding and entry assays. **f**, **g** NCAM1 ECD protein and anti-NCAM1 antibody inhibit ZIKV attachment to U-251 MG cells (**f**). *P = 0.0168; **P = 0.0047. NCAM1 ECD protein and anti-NCAM1 antibody inhibit ZIKV internalized to U-251 MG cells (**g**). Data from four experiments (*n* = 4). NCAM1 ECD protein to mock, **P = 0.0013; Anti-NCAM1 antibody to mock, **P = 0.0026. Significantly different from Mock cells (two-tailed Student’s *t* test). **h**, **i** RT-qPCR revealed an increase in viral attachment (**h**) and entry (**i**) following NCAM1 overexpression in 293T cells. Data from three experiments (**h** *n* = 3; **i** NC treated *n* = 10, NCAM1 treated *n* = 7). *P = 0.0133 (**h**); *P = 0.0195 (**i**). Sgnificantly different from NC (negative control) treated 293T cells (two-tailed Student’s *t* test). **j** Flow cytometry analyses of NCAM1 expression in NCAM1-KO and WT U-251 MG cells. **k** Immunofluorescence (IF) staining analyses of ZIKV infection. Images shown are representative of three independent experiments. Scale bars, 50 μm. **l** Statistical analyses. Each biological replicate (*n* = 4) contains 3000 analyzed cells. **P = 0.0044. Significantly different from WT cells (two-tailed Student’s *t* test). Quantitative data in this figure are shown as the mean ± s.e.m. DAPI, blue; ZIKVE, red; NCAM1, green. Source data are provided as a Source Data file.

Finally, considering NCAM1 was crosslinked at several time points, totally not detected in control samples and NCAM1 is abundantly expressed in brain, we further examined whether NCAM1 is a potential receptor for ZIKV infection leading to neurological disorders. To assess the physiological importance of NCAM1 interaction with ZIKV, we first examined the surface expression of NCAM1 on U-251 MG and Vero cells. We found that NCAM1 was highly expressed on the surface of U-251 MG and Vero cells (Fig. 4a). The immunofluorescence image also revealed that NCAM1 is located on the membrane of the transfected HEK 293T cells (Fig. 4b). To validate the interaction between NCAM1 and ZIKV ENV, we performed co-immunoprecipitation experiments in the context of ZIKV ENV and NCAM1-Flag overexpression. Immunoblot analyses revealed that NCAM1 specifically interacted with the ZIKV ENV (Fig. 4c), but not with EGFR, another transmembrane glycoprotein as a negative control (Fig. 4d). In addition, we found that the other host factor identified in our study, HSPA8, previously reported as a key host factor for ZIKV infection and ZIKV nonstructural protein stabilization^32^, also bound to the ZIKV ENV protein directly (Supplementary Fig. 7b). We knocked out HSPA8 in U-251 MG cells using two different CRISPR/Cas9 sgRNAs. The knockout efficiency by the sgRNAs was confirmed with western blot analysis and the efficiency of sgRNA2 is lower than that of sgRNA1 (Supplementary Fig. 7c). We found that HSPA8 depletion by sgRNA1 in U-251 MG cells remarkably attenuated ZIKV infection (Supplementary Fig. 7c). To further assess the effect of NCAM1 on ZIKV binding and entry, we employed NCAM1 extracellular domain (ECD) protein and anti-NCAM1 antibody to compete and block NCAM1 binding, respectively, and examined whether it could lead to the reduction in ZIKV attachment and internalization. Preincubation with NCAM1 ECD protein, but not the control protein, remarkably reduced ZIKV binding (Fig. 4f) and entry (Fig. 4g) into U-251 MG cells. Similarly, pretreatment of U-251 MG cells with the anti-NCAM1 antibody also reduced ZIKV binding (Fig. 4f) and entry (Fig. 4g). NCAM1 ECD and antibody also inhibited ZIKV binding to Vero cells (Supplementary Fig. 7d), but had no statistical difference on the ZIKV internalization (Supplementary Fig. 7e).

## Discussion

In this study, we present a chemical proteomic approach in which virus was chemically tagged with a biocompatible probe, to reveal the virus–host interactome in real time. The work demonstrates how chemical proteomics can facilitate our understanding of molecular mechanisms and key players in virus infection. Specific identification of virus-interacting proteins was made possible by the integration of several techniques: a multifunctional chemical probe that can achieve the labeling, crosslinking, and isolation steps, photo-reactive crosslinking that allows us to select designated time points and capture interacting proteins covalently to minimize loss during lysis and washing, and label-free MS-based quantitation. In particular, the analyses of proteomic data sets with or without virus infection at different time points enabled the extraction of temporal and spatial information during the virus infection, which provides a useful and universal tool to study any pathogen invasion in theory. Compared with the previously reported mass spectrometric methods for the identification of receptors specific to glycoproteins^10,11^, our method offers the additional advantage of covalently linking proteins at different time points, thus serving the dual purpose of identification of receptors and other host factors involved at different stages of virus entry.

The chemical proteomics strategy was applied to ZIKV and enabled us to identify multiple ZIKV-interacting proteins that indicate ZIKV subcellular localizations and potential entry mechanisms, among which a new ZIKV receptor was discovered and validated through virus attachment and entry assays. Overexpression of NCAM1 in HEK 293T cells increased viral binding and entry. NCAM1 depletion in U-251 MG cells remarkably inhibited ZIKV infection. Inhibition of NCAM1 receptor by NCAM1 ECD and anti-NCAM1 antibody reduced the ZIKV attachment and entry into U-251 MG cells. There was also 40% of reduction in ZIKV attachment to Vero cells but no statistical significant reduction was observed for the ZIKV entry into Vero cells. We reason that Vero cells are less dependent on specific receptors for virus infection and therefore are commonly used as a model cell line for virus infection. On the other hand, ZIKV infection of U-251 MG cells are highly dependent on specific receptors. Once a specific receptor, e.g., NCAM1, was blocked, significant reduction in attachment and entry into U-251 MG cells was observed.

To exclude a general effect of NCAM1 on other viruses binding and entry, we performed an inhibition assay using Influenza A virus (IAV) and dengue virus (DENV-2). NCAM1 ECD and anti-NCAM1 antibody had minimal effects on IAV binding and entry (Supplementary Fig. 7f, left), but NCAM1 ECD had significant effect on DENV binding and anti-NCAM1 antibody had minimal effects on DENV-2 binding and entry (Supplementary Fig. 7f, right), suggesting that NCAM1 may affect the attachment of ZIKV or DENV but not IAV. To further confirm the receptor activity of NCAM1, we overexpressed NCAM1 in HEK 293T cells and the expression efficiency was validated with immunofluorescence imaging (Fig. 4b) and western blotting assays (Supplementary Fig. 8), and then we performed binding and entry assays. Heterologous expression of human NCAM1 in 293T cells enhanced both ZIKV attachment (Fig. 4h) and internalization (Fig. 4i). To further demonstrate the requirement of NCAM1 for infection by ZIKV, we knocked out NCAM1 in U-251 MG cells using two different CRISPR/Cas9 sgRNAs. The knockout efficiency by the sgRNAs was confirmed with flow cytometry staining and the efficiency of sgRNA2 (green) is lower than that of sgRNA1 (blue) (Fig. 4j). We found that NCAM1 depletion by sgRNA1 in U-251 MG cells remarkably attenuated ZIKV infection (Fig. 4k, l). This result supports our observation that NCAM1 could be a potential receptor for ZIKV infection (Supplementary Fig. 7g).

The chemical proteomics strategy highlighted its unique feature that allowed us to track the virus movement in real time, which is challenging due to the highly dynamic nature of the process and the transient virus–host protein interactions. Moreover, the crosslinking chemistry permits the identification of potential receptors which present analytical challenges to identify the interaction on cell membrane. Lastly, this technology can be applied to relatively unstable enveloped viruses, owing to the minimal labeling by cysteine-reactive maleimide group.

## Methods

### Cells and reagents

U-251 MG (human astrocytoma cells; ECACC-08061901), Vero (African green monkey kidney cells; CCL-81, ATCC), and HEK293T (human embryonic kidney cells; CRL-1573, ATCC) were maintained in DMEM media (Gibco) supplemented with heat-inactivated 10% FBS (Gibco), 100 IU/mL of penicillin and 100 µg/mL of streptomycin, at 37 °C and under 5% CO_2_. U-251 MG cell line was purchased from BeNa Culture Collection (BNCC). 293T and Vero cell lines were purchased from the American Type Culture Collection (ATCC). Low passage cells were used for the virus propagation and other infection experiments. The NCAM1 ECD protein (Cat. No. 10673-H08H) for inhibition experiment and the rabbit anti-NCAM1 antibodies (PE) (Cat. no. 10673-MM05-P) for staining of surface expression of NCAM1 were purchased from Sino Biological. The anti-NCAM1 monoclonal antibody for inhibition experiment and immunofluorescence assay was purchased from BD Biosciences (Cat. no. 559043), while the antibody for western blot was obtained from Cell Signaling Technology (Cat. no. 3576). All reagents for synthesis were obtained from Sigma-Aldrich, ChemPep Inc, Peptides International Inc, Novabiochem (EMD Millipore), and Alfa Aesar. pCMV-Zika-ENV (Cat. No. VG40543-UT) and pCMV-Zika-NS1 (Cat. No. VG40544-UT) natural native open reading frame mammalian expression plasmids were purchased from Sino Biological. pcDNA3.1+/C-(K)DYK-NCAM1 (Cat. no. OHu00262), pCMV-GFP-Flag (Cat: AG13105-CF), pCMV-EGFR (Cat: HG10001-UT) and pcDNA3.1+/C-(K)DYK-HSPA8 (Cat. no. OHu27538) mammalian expression plasmids were purchased from GenScript.

### Mature ZIKV preparation

Approximately 1 × 10^9^ Vero-Furin cells^36^ were infected at a multiplicity of infection (MOI) of 0.1 with ZIKV (strain H/PF/2013) at 37 °C^2^. The ZIKV MR766 stock was purchased from ATCC (ATCC^®^ VR-1838™). Virus particles were purified from media collected at 60 and 72 h post infection (hpi) according to Sirohi et al.^2^. Briefly, virus particles were precipitated from the media with 8% polyethylene glycol (PEG) 8000 overnight at 4 °C, pelleted at 10,000 × g for 50 min at 4 °C. Resuspended particles were pelleted through a 24% sucrose cushion, resuspended in 0.5 mL NTE buffer (20 mM Tris pH 8.0, 120 mM NaCl, 1 mM EDTA), and purified with a discontinuous gradient in 5% intervals from 35% to 10% K-tartrate, 20 mM Tris pH 8.0, 1 mM EDTA. Mature virus was extracted from the gradient, concentrated, and buffer exchanged into NTE buffer.

### Plaque assay

The plaque assay was performed as described below. Briefly, virus was diluted serially in the order of ten folds and incubated with monolayers of Vero cells for 1 h at room temperature. Cells were layered with agarose and incubated at 37 °C for 3 days. Plaques were counted following cell staining using neutral red.

### Synthesis and purification of a multifunctional chemical probe

The virus-labeling chemical probe was synthesized on the Rink-Amide-AM-Resin (200–400 mesh) 1% DVB manually, using standard solid phase peptide synthesis approach (Supplementary Fig. 1). A 20% piperidine solution in DMF (N,N-Dimethylformamide) was used to deprotect the fmoc (9-Fluorenylmethoxycarbonyl) groups, while 95% TFA (Trifluoroacetic acid) was used for boc (*tert*-Butoxycarbonyl) group deprotection. HCTU (O-(1H-6-Chlorobenzotriazole-1-yl)−1,1,3,3-tetramethyluronium hexafluorophosphate) was utilized as an activating agent for the carboxyl group on the incoming reactant, in presence of the base NMM (4-methylmorpholine). The synthesis was performed on the 30 µmol scale and using 2.5-fold excess of the reagents compared with the resin. Each step involved the deprotection of amine group, activation of carboxyl group followed by coupling reaction. The excess reagents were removed by thorough washing of beads by DMF. Ninhydrin test was performed after each deprotection and coupling reaction.

The synthesis was performed using the strategy, as previously described^13^. 80 mg (30 µmol) of Rink-Amide-AM-Resin was added to the fritted reaction vessel. Beads were conditioned with DMF for 15 min. The solution was removed by filtration, and 20% piperidine in DMF was added to the beads for fmoc deprotection. The mixture was end-to-end rotated for 30 min, and solution was removed followed by washing of beads with DMF. A reaction mixture of Fmoc-Lys-Biotin-OH (44.60 mg, 75 µmol), HCTU (31.03 mg, 75 µmol), and NMM (16.49 µL, 150 µmol) in DMF was added to the resin, and rotated for 4 h at room temperature. The resin was washed with DMF, and fmoc deprotection steps were repeated. A solution of N-Fmoc-Nʺ-succinyl-4,7,10-trioxa-1,13-tridecanediamine (40.70 mg, 75 µmol), HCTU (31.03 mg, 75 µmol), and NMM (16.49 µL, 150 µmol) in DMF was rotated with the resin for 4 h. Excess reagents were removed, and the resin was washed with DMF. Similarly, Fmoc-Lys(Boc)-OH (35.14 mg, 75 µmol), N-Fmoc-Nʺ-succinyl-4,7,10-trioxa-1,13-tridecanediamine (40.70 mg, 75 µmol), and 6-Maleimidohexanoic acid (15.84 mg, 75 µmol) were coupled to the resin after fmoc deprotection steps. The resin was washed with DMF and dichloromethane, and the molecule was cleaved from the resin using 95:5 mixture of TFA:TIS (Triisopropylsilane) for 1.5 h. The cleavage step also deprotects the boc group, making available a free amine in the product. The crude product was concentrated and HPLC (Agilent 1100) purified using a gradient of 5–85% B (A: 0.1% TFA/H_2_O, B: 0.1% TFA/CH_3_OH) over 30 min on Waters XBridge Prep BEH130 C18 column (5 µm, 10 × 250 mm) to yield **1** (25.68 mg, 19.8 µmol, 66%). MALDI-TOF and NMR were used to characterize the product (Supplementary Fig. 2).

^1^H NMR (500 MHz, DMSO-*d*_6_) *δ* 7.91 (d, *J* = 8.1 Hz, 1H), 7.86–7.71 (m, 4H), 7.66 (q, *J* = 5.9 Hz, 2H), 7.54 (bs, 3H), 7.26–7.18 (m, 1H), 6.91 (s, 2H), 6.89–6.83 (m, 1H), 6.42–6.20 (m, 2H), 4.28–4.16 (m, 1H), 4.10–3.94 (m, 3H), 3.49–3.18 (m, 24H), 3.04–2.85 (m, 11H), 2.70 (dd, *J* = 15, 5.6 Hz, 1H), 2.71–2.61 (m, 2H), 2.49 (d, *J* = 12.4 Hz, 1H), 2.33–2.16 (m, 8H), 1.94 (dt, *J* = 13.4, 7.4 Hz, 4H), 1.68–1.47 (m, 11H), 1.46–1.32 (m, 11H), 1.31–0.99 (m, 11H).

^13^C NMR (126 MHz, DMSO) *δ* 174.1, 172.0, 171.8, 171.6, 171.6, 171.5, 171.1, 162.8, 158.2, 158.0, 134.5, 117.4, 115.1, 112.7, 69.8, 69.6, 68.1, 68.1, 61.1, 59.3, 55.5, 52.4, 37.0, 36.0, 35.9, 35.8, 35.3, 35.2, 31.4, 31.2, 30.8, 30.7, 30.6, 29.4, 29.4, 29.3, 29.0, 28.3, 28.1, 27.8, 26.7, 25.8, 25.4, 24.9, 23.0, 22.5.

A 1297.358 peak in MALDI was observed corresponding to the expected M + H^+^.

The pure maleimide-biotin **1** (20 mg, 15.42 µmol) was dissolved in DMF and reacted with excess NHS-LC-Diazirine (succinimidyl-6-(4,4′-azipentanamido)hexanoate) in phosphate buffer pH 8, for 2 h at room temperature. The product was purified by directly injecting into the HPLC and using similar conditions as described above. The virus-labeling chemical probe **2** was obtained as a white powder (14.06 mg, 9.25 µmol, 60%) and characterized by MALDI-TOF, ^1^H and ^13^C NMR (Supplementary Fig. 3).

^1^H NMR (500 MHz, DMSO-*d*_6_) *δ* 8.00–7.88 (m, 2H), 7.88–7.77 (m, 4H), 7.72 (p, *J* = 5.8 Hz, 3H), 7.28 (p, *J* = 2.7, 2.2 Hz, 1H), 6.99 (s, 2H), 6.94 (s, 1H), 6.45–6.31 (m, 2H), 4.32–4.26 (m, 1H), 4.15–4.03 (m, 3H), 3.54–3.41 (m, 17H), 3.40–3.24 (m, 7H), 3.14–2.92 (m, 16H), 2.81 (dd, *J* = 12.4, 5.1 Hz, 1H), 2.56 (d, *J* = 12.4 Hz, 1H), 2.40 – 2.23 (m, 8H), 2.06–1.96 (m, 6H), 1.96–1.88 (m, 2H), 1.72–1.52 (m, 13H), 1.52–1.40 (m, 10H), 1.40–1.08 (m, 18H), 0.96 (s, 3H).

^13^C NMR (126 MHz, DMSO) *δ* 174.0, 171.8, 171.6, 171.5, 171.5, 171.4, 171.1, 170.5, 162.7, 134.5, 69.8, 69.5, 68.1, 61.1, 59.2, 55.4, 52.6, 52.4, 37.0, 35.8, 35.7, 35.4, 35.2, 35.2, 31.5, 31.4, 30.7, 29.9, 29.8, 29.4, 29.3, 29.2, 28.9, 28.9, 28.2, 28.0, 27.8, 26.1, 25.8, 25.3, 25.1, 24.8, 23.0, 19.3.

MALDI showed a peak at 1492.232 *m*/*z*, corresponding to the M–N_2_ + H^+^. This is due to the loss of N_2_ from diazirine under MALDI conditions.

### BSA labeling/virus labeling

Fifty micrograms BSA or Purified ZIKV was diluted to 500 µL with PBS pH 7, and mixed with the labeling chemical probe in the final concentration of 1 mM (Supplementary Fig. 4b–d). The labeling was carried out by gentle end-to-end rotation in 4 °C overnight. For the infection experiment, virus labeling was initiated a day before the cells reached <90% confluency for infection. The reaction was quenched by adding three times excess of cysteine.

### Virus infection and crosslinking of host proteins

Vero cells were first grown in T-150 flasks in DMEM supplemented with 10% FBS, then passaged to the 15 cm plates and grown to <90% confluency. Cells were washed with cold PBS twice, and cooled down to 4 °C. The labeled virus was diluted in DMEM and added to the cells at MOI of 5. Cells were gently rocked for 1 h in 4 °C, to allow for virus attachment. For the receptor crosslinking, the unbound virus was removed, cells were washed once with cold PBS, and directly exposed to the UV light for 15 min on ice. All the above operations were performed on ice and using cold PBS to minimize any virus entry. To understand the virus internalization mechanism, additionally virus was allowed to enter cells by incubation in 37 °C for 4 or 8 min, following pre-attachment for an hour at 4 °C. Subsequent to UV photo crosslinking, cells were collected by scraping in PBS, and stored in −80 °C until further processing. As a control, cells treated with the labeling chemical probe and exposed to UV were included to account for random crosslinking.

### Sample preparation for LC–MS/MS analysis

Frozen cells were lysed in 1% SDS, 50 mM Tris HCl pH 7.5 supplemented with protease inhibitor on ice, using sonication (10 cycles for 10 s each, with an interval of 10 s). Cell lysates were cleared by centrifugation at 14,000 rpm to pellet down cell debris, and supernatant were used for the biotin-Neutravidin affinity purification. Bicinchoninic acid assay (Thermo Fisher Scientific) was performed for protein quantitation, and the lysates equivalent to 1 mg protein for each sample were reduced and alkylated by boiling at 95 °C, in 10 mM TCEP (Tris(2-carboxyethyl)phosphine) and 40 mM CAA (chloroacetamide) respectively. The lysates were then diluted to 0.1% SDS and rotated with 50 µL preconditioned Neutravidin beads slurry in 4 °C overnight. The beads were washed with 0.1% SDS in 50 mM Tris (pH 8.0), 5 M NaCl, 300 mM glycerol in 50 mM Tris (pH 8.0), and then transferred to the low protein binding Eppendorf tubes, where they were further washed three times with 25 mM ammonium bicarbonate (ABC) buffer pH 8. Two hundred microliters ABC buffer was added to the beads, and proteins were digested on-bead at 37 °C using 2 µg Lys-C for 3 h and 200 ng trypsin for 12 h. The supernatant containing peptides was collected and beads were washed twice with 50 µL ABC buffer, further pooled with the supernatant. Peptides were acidified and desalted using in-house StageTips with SDB-XC (3M). The peptides were dried in SpeedVac before subjecting to LC–MS/MS analysis.

### LC–MS/MS analysis

The peptides were dissolved in 0.1% formic acid and injected into Easy-nLC 1000 (Thermo Fisher Scientific). The peptides were separated on a 45 cm in-house column (360 µm OD × 75 µm ID), packed with C18 resin (2.2 µm, 100 Å, Bischoff Chromatography, Leonberg, Germany) and heated to 60 °C with a column heater (Analytical Sales and Services, Flanders, New Jersey). The mobile phase was comprised of 0.1% formic acid in ultra-pure water (solvent A) and 0.1% formic acid in 80% acetonitrile (solvent B), and the gradient used for separation was 10–30% B over a linear 60 min at a flow rate of 250 nL/min. The EASY-nLC 1000 was connected online to the LTQ-Orbitrap Velos Pro mass spectrometer (Thermo Fisher Scientific) by a nanospray source. Data acquisition was performed in the data-dependent mode, in which a full scan (range from *m*/*z* 350–1800 with a resolution of 30,000 at *m*/*z* 400) was followed by MS/MS scans of top 10 intense ions (normalized collision energy 30%, automatic gain control 3e4, maximum injection time 100 ms) with a dynamic exclusion for 60 s and dynamic list of 500.

### Proteomic data analysis

Raw files were processed with MaxQuant v1.6.6.0, and the label-free quantitation (LFQ) was performed. The raw data were searched against UniProtKB (*Rhesus macaque*) FASTA database (Version July 2018, 45,199 entries) with Andromeda search engine, using default parameters. The first peptide precursor mass tolerance was set at 20 ppm, and MS/MS tolerance at 0.6 Da. Carbamidomethylation was set as a fixed modification for cysteines, while oxidation of methionine and acetylation at N-terminus were selected as variable modifications. Enzyme specificity was set to trypsin with maximum two missed cleavages. The search was performed with 1% false discovery rate at both peptide and protein levels. The identifications were transferred from the sequenced peaks to the unidentified peaks of the same *m*/*z* within a time window of 0.7 min (match between runs) across samples. For LFQ, initially, the LFQ intensities were extracted from the MaxQuant output file. Proteins with valid values in minimum two out of three replicates in at least one group were only considered, and values were imputed for all missing values based on normal distribution. Significant proteins at different time points vs. control (${\mathrm{FC}} \,\ge\, 2$, *t* test ${{p}} \,\le\, 0.01$) were considered as crosslinked proteins, which were further processed by homemade MATLAB program for functional analysis.

For heatmap analysis, the expression value (iBAQ) of total quantified proteins were clustered based on Euclidean distances with average linkage using modified function Clustergram in MATLAB (Version R2019b), and heatmap was also visualized by MATLAB. The color shows that there are great changes from sample to sample, where red is upregulation, green is downregulation, black represents no change, and gray represents non available. Each column in the graph represents an experiment condition, and each row corresponds to a gene. Row of protein were normalized by maximum value of corresponding row. The rows and columns are displayed in the order given by the clustering output trees in the two dimensions.

GO enrichment analysis of the differentially expressed proteins were conducted according to the information from GO databases, each bar in the figure denotes the enrichment score from different sample, and enrichment score was defined as −log10(*p*). *p* value was calculated using the hypergeometric formula as below:

1 $${\boldsymbol{p}} \,=\, 1 \,-\, \mathop {\sum}_{{\boldsymbol{i}} \,=\, 0}^{{\boldsymbol{m}} \,-\, 1} {\frac{{\left( {\begin{array}{*{20}{c}} {\boldsymbol{M}} \\ {\boldsymbol{i}} \end{array}} \right)\left( {\begin{array}{*{20}{c}} {{\boldsymbol{N}} \,-\, {\boldsymbol{M}}} \\ {{\boldsymbol{n}} \,-\, {\boldsymbol{i}}} \end{array}} \right)}}{{\left( {\begin{array}{*{20}{c}} {\boldsymbol{N}} \\ {\boldsymbol{n}} \end{array}} \right)}}},$$

*N* is the number of all identified proteins that can be connected with GO analysis information.

*n* is the number of differential proteins in N.

*M* is the number of proteins that can be connected with a certain GO term.

*m* is the number of differential proteins with certain GO term.

If *p* value below 0.05, we regard this GO term as a significant enrichment of differential proteins.

The information of protein–protein interaction of significant proteins were retrieved by STRING database, and visualized by cytoscape.

### Viral attachment and entry assay

For antibody inhibition experiment, U-251 MG cells in twelve-well plate were preincubated with 20 µg/mL anti-NCAM1 antibody (Cat. no. BD-559043, BD) or control isotype IgG antibody (Cat. no. BD-550617, BD) in DMEM supplemented with 2% FBS for 1 h at 37 °C. Cells were then incubated with purified ZIKV (MOI = 100) on ice for 1 h in the presence of antibody. The supernatant was then removed and the cells were washed three times with cold PBS. Cellular RNA was extracted and purified for test the viral attachment. Otherwise, prewarmed medium was added to the cells to initiate ZIKV internalization. The cells were incubated at 37 °C for additional 40 min and then cellular RNA was extracted and purified. Then RT-qPCR was performed to measure viral entry. For NCAM1 protein inhibition experiment, purified ZIKV (PFU = 10^8^) were preincubated with 20 µg NCAM1 ECD (Sino Biological, Cat. no. 10673-H08H) or control protein for 1 h at 37 °C. The viruses (MOI = 100) were then added to U-251 MG cells in twelve-well plate and incubated on ice for 1 h. The supernatant was then removed and the cells were washed three times with cold PBS. Cellular RNA was extracted and purified for test the viral attachment. Otherwise, prewarmed medium was added to the cells to initiate ZIKV internalization. The cells were incubated at 37 °C for additional 40 min and then cellular RNA was extracted and purified. Then RT-qPCR was performed to measure viral entry.

For NCAM1 overexpression experiment, control pcDNA3.1 plasmid or pcDNA3.1-NCAM1 plasmid were transfected to 293T cells separately. Cells were then incubated with purified ZIKV (MOI = 100) on ice for 1 h after 36 h transfection. The supernatant was then removed and the cells were washed three times with cold PBS. The increase in viral attachment was measured with RT-qPCR. On the other hand, prewarmed medium was added to the cells to initiate ZIKV internalization. The cells were incubated at 37 °C for additional 40 min and then cellular RNA was extracted and purified. Then RT-qPCR was performed to measure internalized ZIKV RNA.

### Immunoprecipitation assay

For co-immunoprecipitation experiments, 293T cells (8 × 10^6^ cells per 10 cm dish) were transiently transfected with 14 µg of pCDNA3.1-NCAM1/ pCDNA3.1-HSPA8 and pCMV-NS1/pCMV-E separately for 48 h using TurboFect Transfection Reagent (Thermo Fisher Scientific). Cells were rinsed twice with cold PBS and were then transferred to clean tubes and lysed in cell lysis buffer for immunoprecipitation supplemented with 1% protease inhibitor cocktail (Cat. no. P8340, Sigma). Cell lysates were incubated with PierceTM Protein A/G Agarose (Cat. no. 20422, Sigma) for 4 h at 4 °C and were then subjected to centrifugation at 10,000 × *g* for 10 min at 4 °C. The supernatant was transferred to a new tube and incubated with 30 µL anti-Flag M2 affinity gel (Cat. no. A2220, Sigma) overnight at 4 °C. The Sepharose samples were centrifuged, washed five times with cell lysis buffer and eluted using 3 × Flag peptide (Cat. no. F4799, Sigma). Then all samples were boiled with SDS loading buffer for 10 min.

### Flow cytometry experiments

Surface expression of NCAM1 was analysed in U-251 MG and Vero cells by staining the cells with rabbit anti-NCAM1 antibodies (PE) (1:100, Cat. no. 10673-MM05-P, Sino Biological) at room temperature for 20 min. Cells were washed three times with PBS supplemented with 2% FBS. All flow cytometry experiments were carried out using an LSRFortessa cell analyser (BD Bioscience). Samples were analysed using FlowJo software version 10 (TreeStar).

### CRISPR-Cas9 knockout assay

Oligos encoding sgRNAs for generating knockout cells using CRISPR-Cas9 were cloned into the lentiCRISPRv2 plasmid (Addgene plasmid, Cat. no. 52961) as previously described^17^. The oligo sequences of the sgRNAs targeting NCAM1 and HSPA8 are listed as follows. NCAM1-sgRNA1: AACGCCAACATCGACGACGC; NCAM1-sgRNA2: ACACCACTGAGATCCGCTGC; HSPA8-sgRNA1: ACAGATGCCAAACGTCTGAT; HSPA8-sgRNA2: CTAGACTGTTACCAATGCTG. LentiCRISPRv2 clones containing the guide sequences were sequenced, purified, and used for lentiviral production. To generate heterogeneous knockout cell populations, U-251 MG cells were infected with the lentiCRISPRv2-derived lentivirus for 36 h and were then reseeded into complete DMEM containing 2 µg/mL puromycin for 14 days to select for transduced cells. Surviving populations derived in this manner were propagated and expanded for using.

### RNA extraction and real time-quantitative polymerase chain reaction (RT-qPCR)

RNA was isolated from mammalian cells (U-251 MG, Vero, and HEK293T) using RNeasy mini kit (Qiagen, Valencia, CA) and normalized based on total RNA amount as determined by NanoDrop™ 2000/2000c Spectrophotometer (Thermo Fisher Scientific). RT-qPCR was performed using SuperScript III Platinum SYBR Green One-Step qPCR Kit w/ROX (Thermo Fisher Scientific) and analyzed on Applied Biosystems 7300 real-time PCR system. The samples were subjected to thermal cycling for 2 min at 50 °C, 10 min at 95 °C, and 40 cycles of 15 s at 95 °C and 1 min at 60 °C, at which point data were collected, and this was followed by dissociation curve analysis. The Ct values obtained were converted to the number of ZIKV RNA molecules using a standard curve generated from in vitro-transcribed viral RNA. ZIKV cDNA clone, used for in vitro transcription, was kindly provided by Shi. For the standard curve, the plasmid containing ZIKV cDNA, was linearized by Cla1 and viral RNA was transcribed using T7 RNA polymerase (New England Biolabs). The DNA template was digested by RNAase-free-DNAase enzymatic treatment for 30 min at 37 °C and the viral RNA was subsequently purified by RNeasy mini kit (Qiagen). RNA concentration and quality were determined by NanoDrop and 10E10 copies of RNA were serially diluted ten-fold and subjected to thermal cycling as described above to obtain the standard curve and PCR efficiency. All PCR primers are listed as follows: ZIKV-F1 TGGGAGGTTTGAAGAGGCTG; ZIKV-R1 TCTCAACATGGCAGCAAGATCT; GAPDH-FCTGGGCTACACTGAGCACC; GAPDH-RAAGTGGTCGTTGAGGGCAATG; DENV-F1AAGGACTAGAGGTTAGAGGAGAC; DENV-R1 GGCGTTCTGTGCCTGGAATGAT; IAV-F1 CGCACAGAGACTTGAGGATG; IAV-R1TGGGTCTCCATTCCCATTTA.

### Immunofluorescence

HEK293T cells (for transfection with NCAM1) were seeded on cover slips in 24-well plate. Cells were washed with PBS and fixed with 3.7% paraformaldehyde (PFA) for 10 min at room temperature. Cells were again washed with PBS three times and blocked with 2% BSA in PBS for 1 h. Anti-NCAM1 antibody in blocking solution was incubated with the cells for 1 h at room temperature. Cells were washed three times and incubated with anti-mouse FITC or anti-mouse Alexa Fluor 488 for 1 h at room temperature. DAPI staining was performed for 10 min, followed by final three PBS washes. Cover slips were mounted on glass slide and images were captured using Olympus IX81 fluorescence microscope with a 60X oil immersion objective.

For detecting ZIKV, cells on slides were fixed with 4% PFA for 20 min at room temperature, permeabilized with 0.1% Triton-X 100 in PBS for 5 min, and blocked with blocking buffer (1% BSA and 2% donkey serum diluted in PBS) for 30 min. Immunofluorescence analyses of ZIKV-infected cells were performed using a mouse anti-flavivirus envelop protein antibody (1:300, clone D1-4G2-4-15, Millipore), with a Alexa Fluor 568 donkey anti-mouse IgG (H + L) (1:1000, ab175472, Abcam) as the secondary antibody. All cells were mounted with ProLongTM Gold Antifade with DAPI (Life Technologies, P36931) and imaged with a TissueFAXS 200 flow-type tissue cytometer (TissueGnostics GmbH, Vienna, Austria). All statistical analyses of immunofluorescence staining present the results from at least 3000 cells per replicate, and data are shown as the mean ± s.e.m.

### Western blot

Following overexpression of NCAM1 in HEK293T, cells were lysed at 48 h post transfection. The samples were boiled at 95 °C in gel loading buffer and 1,4-Dithiothreitol (DTT) for 5 min. The cell lysates were separated on the precast NuPAGE 4-12% Bis-Tris polyacrylamide gels (Invitrogen) for 90 min at constant voltage of 150 V. A MOPS solution (50 mM MOPS, 50 mM Tris-base, 1 mM EDTA, 0.1% SDS) was used as a running buffer. The proteins were transferred onto polyvinylidene fluoride membranes in Bicine-Bis-Tris transfer buffer containing 12% methanol, for 75 min at a constant current of 275 mA. The membrane was blocked with 2% BSA in TBST, and probed with anti-human NCAM1 (1:000, 99746, Cell Signaling Technology) for 1 h at room temperature. Following washings, anti-mouse IgG HRP-conjugated secondary antibody (1:5000, 7076 s, Cell Signaling Technology) was utilized for visualization (Supplementary Fig. 8). Western blot detection of ZIKV ENV was performed using a rabbit anti-ENV antibody (1:2000, GTX133314, GeneTex) and anti-NS1 (1:2000, GTX133307, GeneTex) with a goat anti-rabbit IgG-HRP antibody (1:3000, B2615, Santa Cruz Biotechnology) as the secondary antibody. Western blot detection of Flag, EGFR, and GAPDH were performed using an anti-Flag M2 (1:2000, F1804, Sigma), anti-EGFR(1:1000, A11351, ABclonal), and an anti-GAPDH (1:1000, ABclonal) with a goat anti-mouse IgG-HRP (1:5000, 31430, Invitrogen) as the secondary antibody.

### Reporting summary

Further information on research design is available in the Nature Research Reporting Summary linked to this article.

## Supplementary information

Supplementary Information

Description of Additional Supplementary Files

Supplementary Data 1

Supplementary Data 2

Reporting Summary

## Data Availability

The source data underlying Figs. 4a, c, d, f–j, l, 6d, and Supplementary Figs. 4d,  7a–f are provided as a Source Data file. The proteomics data have been deposited to the ProteomeXchange Consortium via the PRIDE^38^ partner repository with the dataset identifier PXD020119 [http://proteomecentral.proteomexchange.org/cgi/GetDataset?ID=PXD020119]. All other data are available from the corresponding authors on reasonable request. Source data are provided with this paper.

## Source data

Source Data

## Code availability

The MATLAB code used for functional analysis has been uploaded to PRIDE partner repository with the dataset identifier PXD020119 [http://proteomecentral.proteomexchange.org/cgi/GetDataset?ID=PXD020119]. Source data are provided with this paper.
