# Supplementary material for: Chemical proteomics tracks virus entry and uncovers NCAM1 as Zika virus receptor
Source: Nat Commun. 2020 Aug 4;11:3896. doi: 10.1038/s41467-020-17638-y (PMC7403387; doi:10.1038/s41467-020-17638-y)
Supplement: Supplementary file 1 — Supplementary Information [file 41467_2020_17638_MOESM1_ESM.pdf]

SUPPLEMENTARY INFORMATION

Chemical Proteomics Tracks Virus Entry and Uncovers NCAM1 As Zika Virus Receptor

Mayank Srivastava[1]†, Ying Zhang[2,3]†*, Jian Chen[4]†, Devika Sirohi[5,6], Andrew Miller[5,6], Yang Zhang[2], Zhilu Chen[2,4], Haojie Lu[2], Jianqing Xu[2,4*], Richard J. Kuhn[5,6*] and W. Andy Tao[1,3,6*]

[1]Department of Chemistry, Purdue University, West Lafayette, IN 47907. USA

[2]Institutes of Biomedical Sciences and NHC Key Laboratory of Glycoconjugates Research, Fudan University, Shanghai 200032. P.R. China

[3]Department of Biochemistry, Purdue University, West Lafayette, IN 47907. USA

[4]Shanghai Public Health Clinical Center, Fudan University, Shanghai 200032. P R. China

[5]Department of Biological Sciences, Purdue University, West Lafayette, IN 47907. USA

[6] Purdue Institute of Inflammation, Immunology and Infectious Disease, Purdue University, West Lafayette, IN 47907. USA

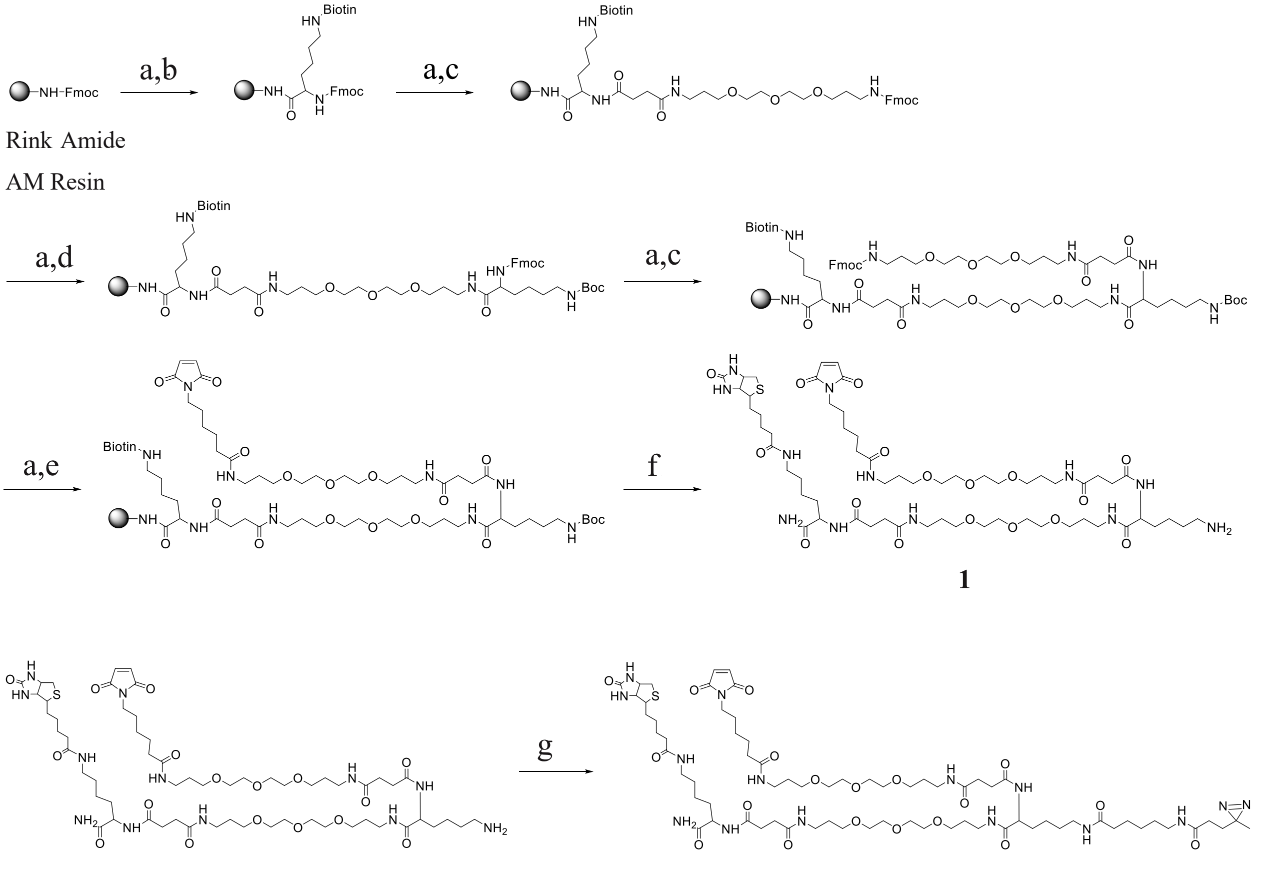

Supplementary Figure 1. Synthesis route of Virus Labeling Chemical probe.[a-g]

Chemical probes and conditions: a 20% Piperidine/DMF; b Fmoc-Lys(Biotin)-OH, HCTU, NMM, DMF; c N-Fmoc-N"-succinyl-4,7,10-trioxa-1,13-tridecanediamine, HCTU, NMM, DMF; d Fmoc-Lys(Boc)-OH, HCTU, NMM, DMF; e 6-Maleimidohexanoic acid, HCTU, NMM, DMF; f TFA/TIS 95:5; g succinimidyl-6-(4,4´-azipentanamido)hexanoate, Phosphate buffer, pH 8.

a

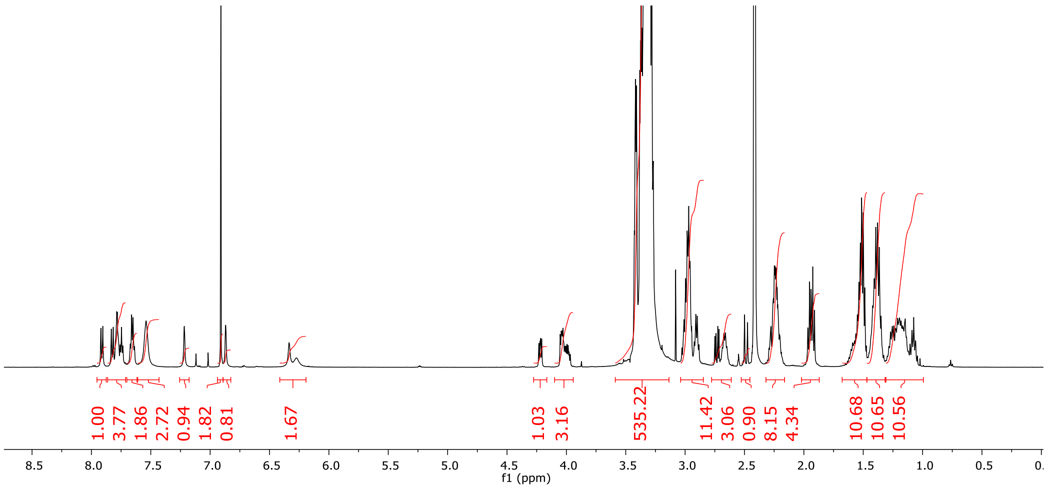

b

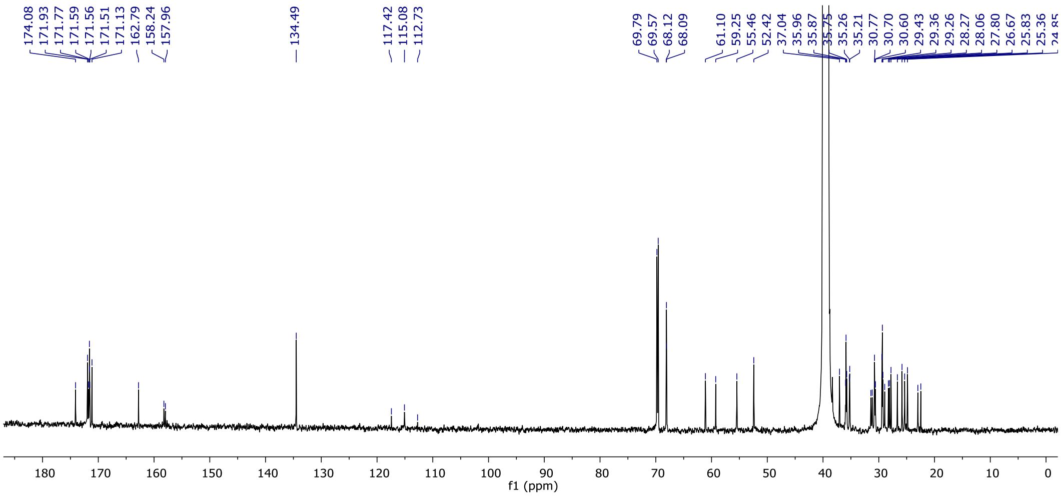

c

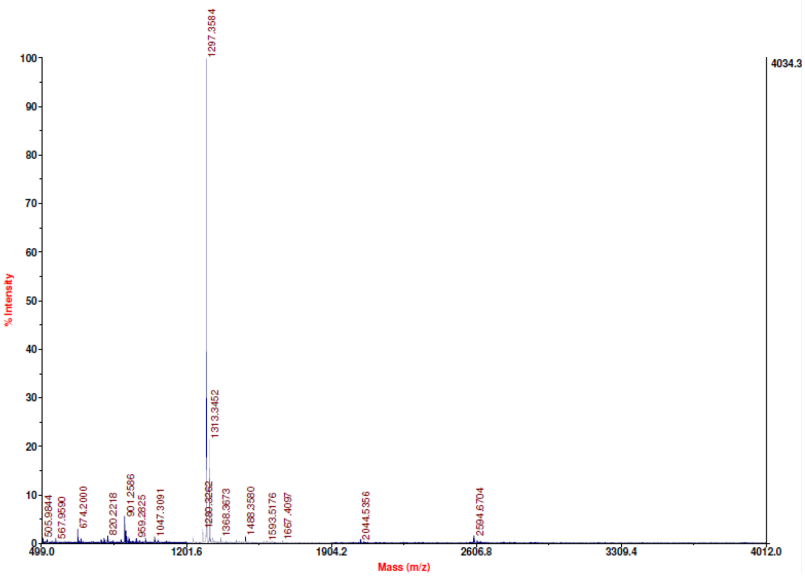

Supplementary Figure 2. Characterization of the probe. a ^{1}H NMR. **b** ^{13}C NMR. and **c** MALDI for compound **1**

a

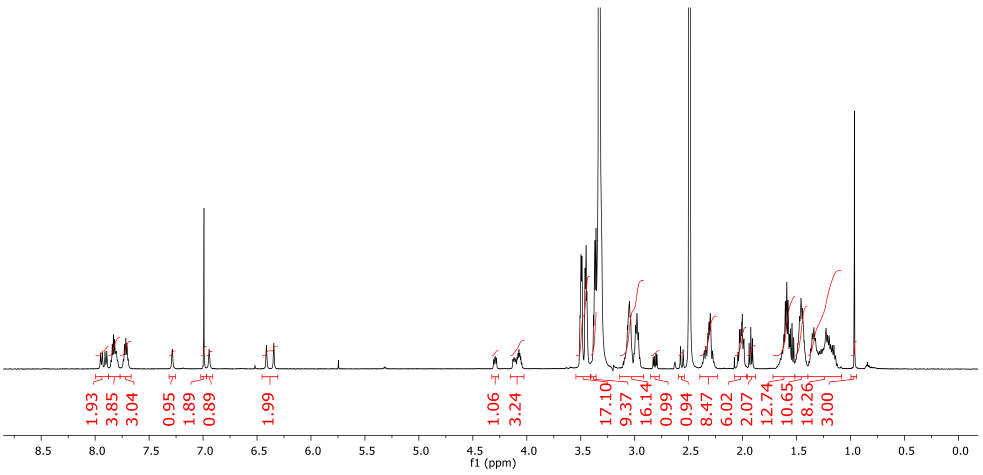

b

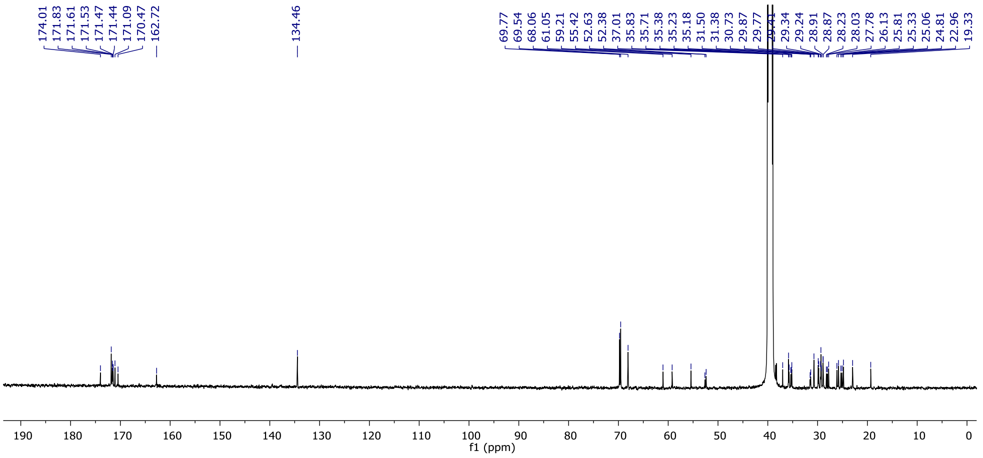

c

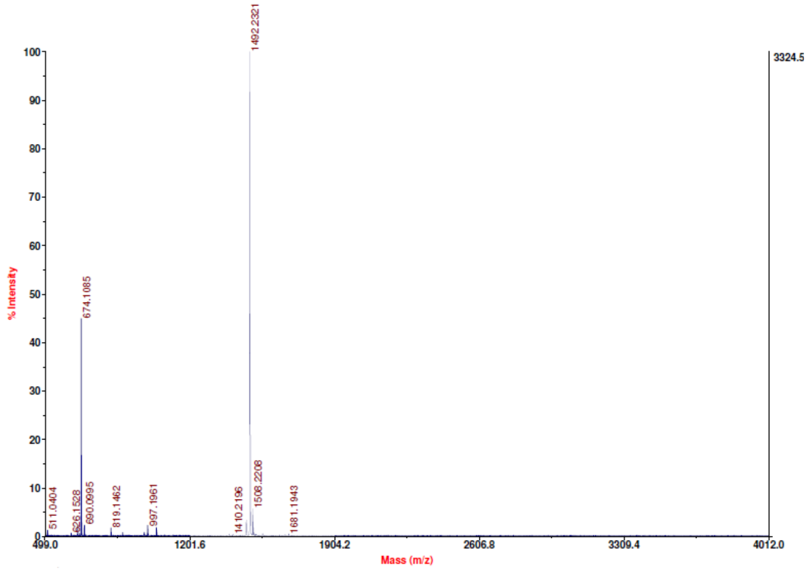

Supplementary Figure 3. Characterization of the probe. a ^{1}H NMR. **b** ^{13}C NMR, and **c** MALDI for compound **2.**

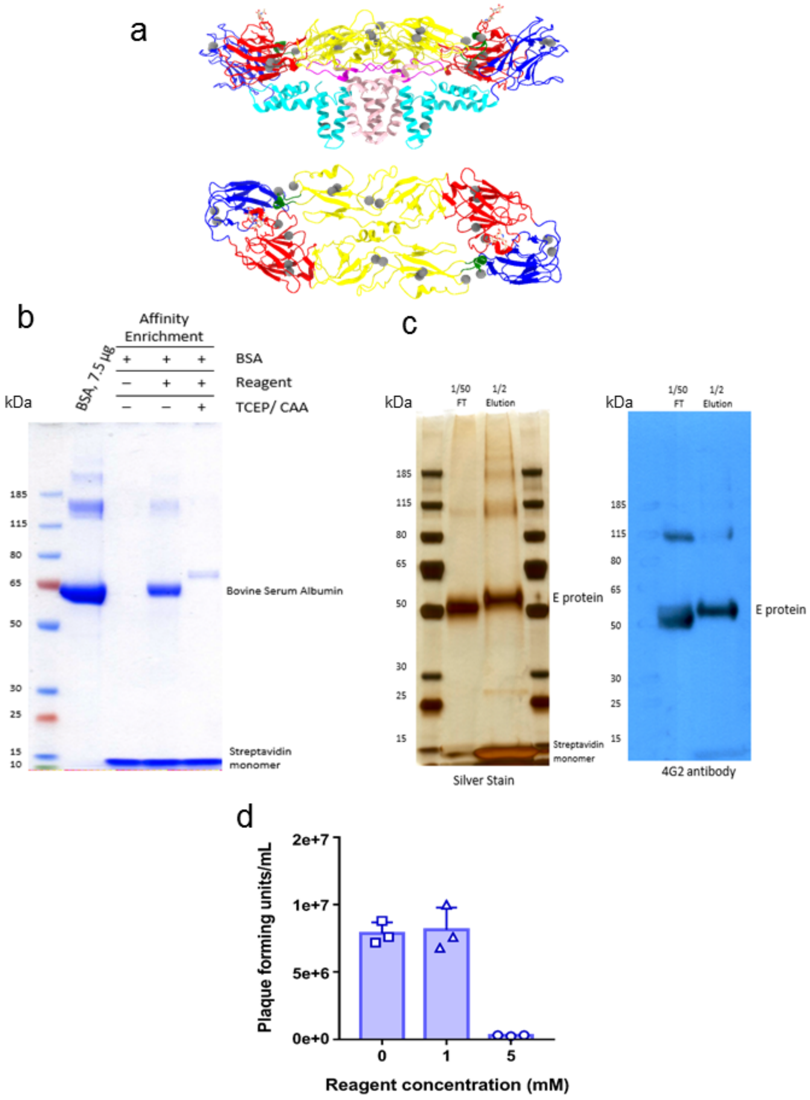

Supplementary Figure 4. Virus labeling by the chemical probe. a Cysteine residues mapped on the surface proteins of ZIKV. Lateral view of a dimer of E-M heterodimer. 90 such dimers form the icosahedral ZIKV surface (Upper panel). Top view of E dimer showing only the ectodomain. The E protein domains are colored as per convention, with domain I, II, and III in red, yellow, and blue, respectively and the fusion loop in green. The E stem/transmembrane domains are depicted in cyan. The M protein ectodomain and stem/transmembrane domains are displayed in magenta and light pink,

respectively. The glycan on E (Asn 154) is shown in stick representation. The C-alpha atoms of the 13 cysteine residues on each E monomer (at position 3, 30, 60, 74, 92, 105, 116, 121, 190, 291, 308, 339 and 488) are represented as grey spheres (Lower panel). **b** Bovine Serum Albumin (BSA) was labeled with the chemical probe in phosphate buffer pH 7 overnight. The reaction was quenched with excess cysteine, and the labeled proteins were enriched on streptavidin beads. The chemical probe labeling efficiency was quantified by comparing the protein band intensity for labeled BSA with the amount started with. A no-chemical probe control was employed to account for non-specific binders. In parallel, the BSA was pre-reduced and alkylated prior to labeling, to confirm cysteine as the labeling site on the protein by the maleimide-diazirine-biotin reagent. **c** Purified ZIKV was labeled by the chemical probe using the same protocol as above. Silver stain and western blot (4G2, anti-E) demonstrating successful labeling of E proteins of ZIKV. **d** The infectivity of labeled virus was confirmed by plaque assay. The number of plaque forming units (pfu) after virus before and labeling with different amount of the chemical probe were measured. Data in this figure are from three replicates with mean ± s.e.m (n=3). Source data are provided as a Source Data file.

IRCIGVSNRDFVEGMSGGTWVDVVLEHGGCVTVMAQDKPTVDIELVTTTVSNMAEVRSYCY
EASISDMASDSRCPTQGEAYLDKQSDTQYVCKRTLVDRGWGNGCGLFGKGSLVTCAKFTCS
KKMTGKSIQPENLEYRIMLSVHGSQHSGMIGYETDEDRAKVEVTPNSPRAEATLGGFGSLGL
DCEPRTGLDFSDLYYLTMNNKHWLVHKEWFHDIPLPWHAGADTGTPHWNNKEALVEFKDA
HAKRQTVVVLGSQEGAVHTALAGALEAEMDGAKGRLFSGHLKCRLKMDKLRLKGVSYSLC
TAAFTFTKVPAETLHGTVTVEVQYAGTDGPCKIPVQMAVDMQTLTPVGRLITANPVITESTEN
SKMMLELDPPFGDSYIVIGVGDKKITHHWHRSGSTIGKAFEATVRGAKRMAVLGDTAWDFG
SVGGVFNSLGKGIHQIFGAAFKSLFGGMSWFSQILIGTLLVWLGLNTKNGSISLTCLALGGVMI
FLSTAVSA

Supplementary Figure 5. Mass spectrometric identification of peptides from E protein of ZIKV. Peptides identified by mass spectrometry are marked in grey.

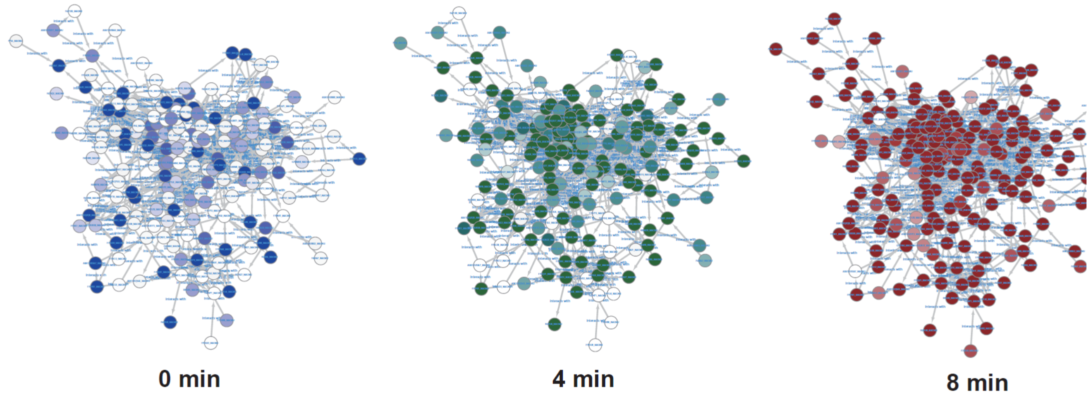

Supplementary Figure 6. Network analysis using STRING showing the protein-protein interactions at different time points of infection. Visualization was performed by Cytoscape. The interactions (or edges) were shown in different colors for the three time points: Blue for 0 mins, Green for 4 mins, and Red for 8 mins of ZIKV infection.

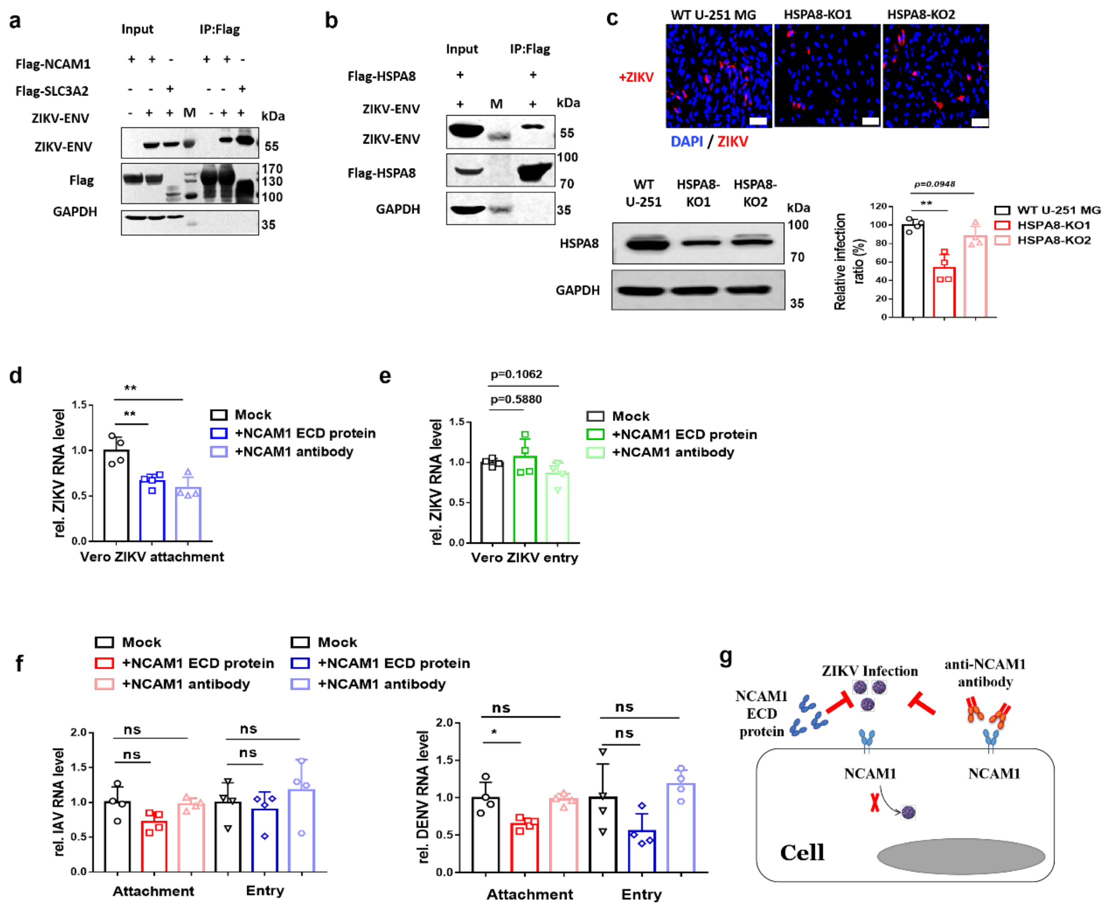

Supplementary Figure 7. Identification of NCAM1 as a potential receptor for ZIKV. a-b,= Immunoblot (IB) and immunoprecipitation (IP) of lysates of ZIKV-ENV and Flag-SLC3A2 (a) or Flag-HSPA8 (b) overexpressed 293T cells. **c** Immunofluorescence (IF) staining analyses and statistical analyses of ZIKV infection. Images shown are representative of three independent experiments. Scale bars, 50 µm. Each biological replicate (n=4) contains 3000 analysed cells. **, P=0.0011. Significantly different from WT cells (two-tailed Student's t-test). **d-e** NCAM1 ECD protein and anti-NCAM1antibody inhibit ZIKV attachment to Vero cells (d). NCAM1 ECD protein and anti-NCAM1 antibody have no effect on ZIKV internalization to Vero cells (e). Data from four experiments (n=4). NCAM1 ECD protein to mock, **, P=0.0071; Anti-NCAM1 antibody to mock, **, P=0.0052. Significantly different from

Mock cells (two-tailed Student's t-test). **f** NCAM1 ECD protein and anti-NCAM1 antibody have no effect on IAV (left) and minimal effect on DENV-2 (right) attachment and no effect on internalization to U-251 MG cells. Data from four experiments (n=4). *, P=0.0197; ns, none significance. Significantly different from Mock cells (two-tailed Student’s t-test). **g** Model for the function of NCAM1 in ZIKV infection, highlighting the role of NCAM1 in ZIKV binding and entry. Quantitative data in this figure are shown as the mean±s.e.m. DAPI, blue; ZIKVE, red.

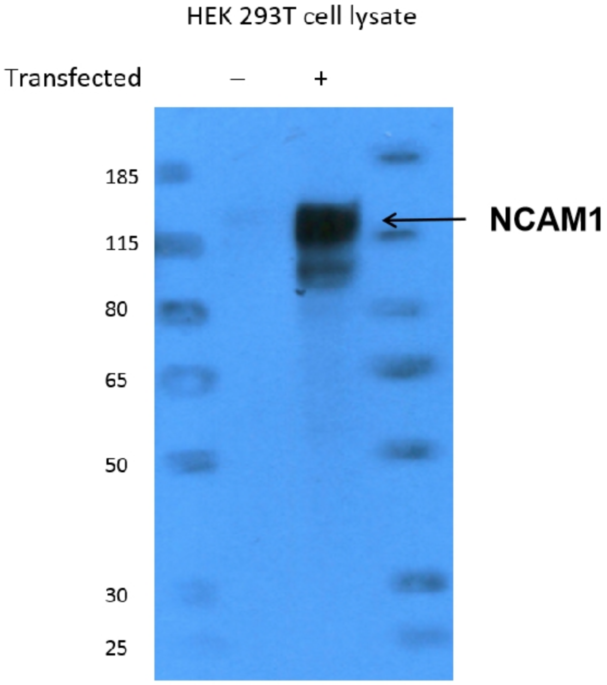

Supplementary Figure 8. Western blot for overexpressed NCAM1 in HEK293T cells. 48 hours post transfection, cells were collected and lysed, and probed against anti-NCAM1 followed by anti-mouse IgG HRP-conjugated secondary antibody. A strong signal was observed in 10 µg lysate after NCAM1 overexpression, while no significant band was observed for 10 µg of non-transfected HEK293T cell lysate.
