# Supplementary material for: Chemical proteomics tracks virus entry and uncovers NCAM1 as Zika virus receptor
Source: Nat Commun. 2020 Aug 4;11:3896. doi: 10.1038/s41467-020-17638-y (PMC7403387; doi:10.1038/s41467-020-17638-y)
Supplement: Supplementary file 2 — Description of Additional Supplementary Files [file 41467_2020_17638_MOESM2_ESM.docx]

**SUPPLEMENTARY DATA LEGENDS**

**Supplementary Data 1:** List of crosslinked proteins at different time points

**Supplementary Data 2:** List of highlighted proteins in proposed endocytosis pathways of ZIKV entry into host cells.
