# Supplementary material for: Chemical proteomics tracks virus entry and uncovers NCAM1 as Zika virus receptor
Source: Nat Commun. 2020 Aug 4;11:3896. doi: 10.1038/s41467-020-17638-y (PMC7403387; doi:10.1038/s41467-020-17638-y)
Supplement: Supplementary file 5 — Reporting Summary [file 41467_2020_17638_MOESM5_ESM.pdf]

nature research

Corresponding author(s): W. Andy Tao

Last updated by author(s): Jul 7, 2020

Reporting Summary

Nature Research wishes to improve the reproducibility of the work that we publish. This form provides structure for consistency and transparency in reporting. For further information on Nature Research policies, see our Editorial Policies and the Editorial Policy Checklist.

Statistics

For all statistical analyses, confirm that the following items are present in the figure legend, table legend, main text, or Methods section.

n/a	Confirmed	
☐	☒	The exact sample size (*n*) for each experimental group/condition, given as a discrete number and unit of measurement
☐	☒	A statement on whether measurements were taken from distinct samples or whether the same sample was measured repeatedly
☐	☒	The statistical test(s) used AND whether they are one- or two-sided<br>*Only common tests should be described solely by name; describe more complex techniques in the Methods section.*
☐	☒	A description of all covariates tested
☒	☐	A description of any assumptions or corrections, such as tests of normality and adjustment for multiple comparisons
☐	☒	A full description of the statistical parameters including central tendency (e.g. means) or other basic estimates (e.g. regression coefficient) AND variation (e.g. standard deviation) or associated estimates of uncertainty (e.g. confidence intervals)
☐	☒	For null hypothesis testing, the test statistic (e.g. *F*, *t*, *r*) with confidence intervals, effect sizes, degrees of freedom and *P* value noted<br>*Give P values as exact values whenever suitable.*
☒	☐	For Bayesian analysis, information on the choice of priors and Markov chain Monte Carlo settings
☒	☐	For hierarchical and complex designs, identification of the appropriate level for tests and full reporting of outcomes
☐	☒	Estimates of effect sizes (e.g. Cohen's *d*, Pearson's *r*), indicating how they were calculated

Our web collection on statistics for biologists contains articles on many of the points above.

Software and code

Policy information about availability of computer code

Data collection	Thermo Xcalibur 3.0.63; LSRFortessa cell analyser (BD Bioscience);TissueFAXS 200 flow-type tissue cytometer (TissueGnostics GmbH, Vienna, Austria)
Data analysis	MaxQuant v1.6.6.0; Clustergram in MATLAB R2019b; FlowJo 10

For manuscripts utilizing custom algorithms or software that are central to the research but not yet described in published literature, software must be made available to editors and reviewers. We strongly encourage code deposition in a community repository (e.g. GitHub). See the Nature Research guidelines for submitting code & software for further information.

Data

Policy information about availability of data

All manuscripts must include a data availability statement. This statement should provide the following information, where applicable:

- Accession codes, unique identifiers, or web links for publicly available datasets
- A list of figures that have associated raw data
- A description of any restrictions on data availability

The source data underlying Figs. 4a, c, d, f-j, l; 6d, and Supplementary Figs. 4d and Figs.7a-f are provided as a Source Data file. The mass spectrometry proteomics data as well as the the MATLAB code used for functional analysis have been deposited to the ProteomeXchange Consortium via the PRIDE partner repository with the dataset identifier PXD020119.

Field-specific reporting

Please select the one below that is the best fit for your research. If you are not sure, read the appropriate sections before making your selection.

☒ Life sciences ☐ Behavioural & social sciences ☐ Ecological, evolutionary & environmental sciences

For a reference copy of the document with all sections, see nature.com/documents/nr-reporting-summary-flat.pdf

Life sciences study design

All studies must disclose on these points even when the disclosure is negative.

Sample size	Experiments were performed using sample sizes based on standard protocols in the field which allow for statistical determination of changes between given samples.
Data exclusions	No data were excluded.
Replication	Three or more replicates were used for each sample. All replicates were successful.
Randomization	Each plate of cells with similar confluency, was randomly assigned to different groups.
Blinding	The investigators were not blinded to group allocation during data collection and analysis. The blinding was not possible because of different incubation times of the cells with virus for different samples.

Reporting for specific materials, systems and methods

We require information from authors about some types of materials, experimental systems and methods used in many studies. Here, indicate whether each material, system or method listed is relevant to your study. If you are not sure if a list item applies to your research, read the appropriate section before selecting a response.

Materials & experimental systems

n/a	Involved in the study	
☐	☒	Antibodies
☐	☒	Eukaryotic cell lines
☒	☐	Palaeontology and archaeology
☒	☐	Animals and other organisms
☒	☐	Human research participants
☒	☐	Clinical data
☒	☐	Dual use research of concern

Methods

n/a	Involved in the study	
☒	☐	ChIP-seq
☐	☒	Flow cytometry
☒	☐	MRI-based neuroimaging

Antibodies

Antibodies used	Primary antibodies: anti-NCAM1 antibody (Cat. No. BD-559043, 20 µg/mL), control isotype IgG antibody (Cat. No. BD-550617), rabbit anti-NCAM1 antibodies (PE) (FACS-1:100, Cat. No. 10673-MM05-P, Sino Biological) ,mouse anti-flavivirus envelop protein antibody (IF-1:300, clone D1-4G2-4-15, Millipore), anti-human NCAM1 (WB-1:1000, 99746, Cell Signaling), rabbit anti-ENV antibody (WB-1:2000, GTX133314, GeneTex) , anti-Flag M2 (WB-1:2000,F1804, Sigma), anti-EGFR(WB-1:1000, A11351, ABclonal) and an anti-GAPDH (WB-1:1000, ABclonal), anti-HSPA8 (WB-1:1000, A14001,ABclonal).<br>Secondary antibodies: goat anti-rabbit IgG-HRP antibody (WB-1:3000, B2615, Santa Cruz Biotechnology) , goat anti-mouse IgG-HRP (WB-1:5000, 31430, Invitrogen), Alexa Fluor 568 donkey anti-mouse IgG (H+L) (IF-1:1000, ab175472, Abcam).
Validation	All the antibodies used in this study were validated either by the comercial source or the provided reference for the applications used in this manuscript.

Eukaryotic cell lines

Policy information about cell lines

Cell line source(s)	U-251 MG (ECACC-08061901); Vero (CCL-81, ATCC); and HEK293T (CRL-1573, ATCC)
Authentication	Cell lines were not authenticated.
Mycoplasma contamination	All cell lines were tested negative for mycoplasma contamination.

Commonly misidentified lines (See ICLAC register)	No misidentified cell lines used in this study.

Flow Cytometry

Plots

Confirm that:

- [x] The axis labels state the marker and fluorochrome used (e.g. CD4-FITC).
- [x] The axis scales are clearly visible. Include numbers along axes only for bottom left plot of group (a 'group' is an analysis of identical markers).
- [x] All plots are contour plots with outliers or pseudocolor plots.
- [ ] A numerical value for number of cells or percentage (with statistics) is provided.

Methodology

Sample preparation	Sample preparation listed in Methods
Instrument	LSRFortessa cell analyser (BD Bioscience)
Software	FlowJo software version 10 (TreeStar)
Cell population abundance	Cell population abundance was available on Figure 4a and j.
Gating strategy	Gating strategy was available on Figure 4a and j.

- [x] Tick this box to confirm that a figure exemplifying the gating strategy is provided in the Supplementary Information.
